# A Relational Agent Intervention for Adolescents Seeking Mental Health Treatment: Protocol for a Randomized Controlled Trial

**DOI:** 10.2196/44940

**Published:** 2023-03-03

**Authors:** Emil Chiauzzi, Athena Robinson, Kate Martin, Carl Petersen, Nicole Wells, Andre Williams, Mary Margaret Gleason

**Affiliations:** 1 Woebot Health San Francisco, CA United States; 2 Children's Hospital of The King's Daughters Norfolk, VA United States

**Keywords:** adolescent, digital health, cognitive behavioral therapy, CBT, chatbot, feasibility, therapeutic alliance, depression, anxiety, relational agent, mental health care, intervention, agent, youth, teenager, mental health, treatment, protocol, feasibility, randomized controlled trial, acceptability, telehealth, outcome, utility

## Abstract

**Background:**

Unmet pediatric mental health (MH) needs are growing as rates of pediatric depression and anxiety dramatically increase. Access to care is limited by multiple factors, including a shortage of clinicians trained in developmentally specific, evidence-based services. Novel approaches to MH care delivery, including technology-leveraged and readily accessible options, need to be evaluated in service of expanding evidence-based services to youths and their families. Preliminary evidence supports the use of Woebot, a relational agent that digitally delivers guided cognitive behavioral therapy (CBT) through a mobile app, for adults with MH concerns. However, no studies have evaluated the feasibility and acceptability of such app-delivered relational agents specifically for adolescents with depression and/or anxiety within an outpatient MH clinic, nor compared them to other MH support services.

**Objective:**

This paper describes the protocol for a randomized controlled trial evaluating the feasibility and acceptability of an investigational device, Woebot for Adolescents (W-GenZD), within an outpatient MH clinic for youths presenting with depression and/or anxiety. The study’s secondary aim will compare the clinical outcomes of self-reported depressive symptoms with W-GenZD and a telehealth-delivered CBT-based skills group (CBT-group). Tertiary aims will evaluate additional clinical outcomes and therapeutic alliance between adolescents in W-GenZD and the CBT-group.

**Methods:**

Participants include youths aged 13-17 years with depression and/or anxiety seeking care from an outpatient MH clinic at a children’s hospital. Eligible youths will have no recent safety concerns or complex comorbid clinical diagnoses; have no concurrent individual therapy; and, if on medications, are on stable doses, based on clinical screening and as well as study-specific criteria.

**Results:**

Recruitment began in May 2022. As of December 8, 2022, we have randomized 133 participants.

**Conclusions:**

Establishing the feasibility and acceptability of W-GenZD within an outpatient MH clinical setting will add to the field’s current understanding of the utility and implementation considerations of this MH care service modality. The study will also evaluate the noninferiority of W-GenZD against the CBT-group. Findings may also have implications for patients, families, and providers looking for additional MH support options for adolescents seeking help for their depression and/or anxiety. Such options expand the menu of supports for youths with lower-intensity needs as well as possibly reduce waitlists and optimize clinician deployment toward more severe cases.

**Trial Registration:**

ClinicalTrials.gov NCT05372913; https://clinicaltrials.gov/ct2/show/NCT05372913

**International Registered Report Identifier (IRRID):**

DERR1-10.2196/44940

## Introduction

### Background

In 2021, leading child-focused organizations declared a national emergency in child and adolescent mental health (MH) [1], echoed by the Surgeon General’s advisory on youth MH [2]. The prevalence of MH concerns among youths has increased exponentially since the onset of the COVID-19 pandemic [1], with recent estimates indicating a doubling of clinically elevated depression and anxiety symptoms [3]. Depression onset in adolescence confers risk for long-term depression as well as other psychiatric sequelae [4,5]. Similarly, persistent untreated anxiety in adolescence can interfere with typical development, potentially affecting the foundation for longer-term well-being, resilience, and functioning [6]. The World Health Organization states that depression and anxiety are the leading cause of illness and disability among adolescents [7], but only 41.6% of adolescents with a major depressive episode were treated in the past year [8]*.* Lack of access to child MH clinicians skilled in high-quality, evidence-based psychotherapy is an unfortunately common and familiar treatment barrier for many adolescents [9]. The rising incidence of MH concerns coupled with unmet needs for care underscores that innovative alternative approaches for adolescent MH care are urgently needed.

“Stepped care,” a MH service delivery approach recommended by the National Institute for Health and Care Excellence, suggests that individuals (including youths) with lower-acuity symptoms initially receive low-intensity services, such as guided self-help, bibliotherapy, or brief therapeutic or supportive interventions, as the first “step” in their care [10]. Those with initial higher-acuity or persistent symptoms after low-intensity intervention receive more intensive services. Stepped care models attempt to distribute limited clinical intervention resources optimally [10]. Early low-intensity intervention options may include technology modalities such as smartphone app-delivered interventions. Integrating such technology-leveraged treatment options within a health care ecosystem increases the total continuum of service offerings and may reduce waitlists as well optimize clinician deployment toward more severe cases.

### Technology-Leveraged MH Care Interventions

Decades of research and treatment guidelines suggest that cognitive behavioral therapy (CBT) is the treatment of choice for adolescents with depression and/or anxiety [11,12]. Indeed, CBT is efficacious when delivered in guided self-help and group-based formats [13,14] or via telehealth [15]. Computer-delivered, rather than clinician-delivered, CBT programs are also effective among an adolescent population [16]. However, these programs suffer from poor adherence, as well as limited tailoring and interactional styles typical in CBT treatment [17,18]. Recently, the availability of MH applications for youths, including video games, mood trackers, journals, and text-based solutions [16], has grown exponentially, yet data on their clinical efficacy remain limited. More meaningful interactions may be possible through the use of interactive technology, such as conversational agents. This may be particularly advantageous in adolescent MH interventions, as this group is well-versed in conversational agent and texting interactions [19]. Such interactions may improve users’ treatment responsiveness to interventions [20,21].

Woebot for Adolescents (W-GenZD) is an investigational mobile app with a relational agent, “Woebot,” that offers a CBT-guided self-help program designed to reduce symptoms of depression and/or anxiety. Although primarily based in CBT, W-GenZD also includes elements of other well-researched and scientifically validated psychotherapeutic interventions including interpersonal psychotherapy for adolescents and dialectical behavior therapy [22,23]. Previous research on other Woebot programs have demonstrated feasibility, acceptability, and preliminary efficacy among various populations including young adults with depression [18], adults with substance abuse concerns [24], and adult women with postpartum mood concerns [25]. A retrospective analysis with over 35,000 adults found that users reported forming a therapeutic alliance with Woebot [20]. Therapeutic alliance, a construct familiar in psychotherapeutic treatment outcomes research, refers to the therapeutic relationship and affective bond between provider and patient and is associated with the efficacy of psychotherapy [26,27]. A randomized controlled pilot among adolescents aged 13-17 years (n=17) demonstrated W-GenZD’s preliminary feasibility and utility in an outpatient primary care pediatric setting [28], but further evaluation of its feasibility and acceptability as well as clinical efficacy is warranted.

The proposed study (trial registration: ClinicalTrials.gov NCT05486611) seeks to understand the feasibility and acceptability of W-GenZD among adolescents seeking treatment for depression and anxiety. The study will also compare 2 forms of technology-leveraged interventions, W-GenZD and brief telehealth-delivered CBT-based skills group (CBT-group), among adolescents presenting with depression and/or anxiety. Both interventions are conceptualized as potential “first-step” interventions within a stepped care model. The setting for the study is an outpatient children’s hospital that currently deploys a brief telehealth-delivered CBT-group as a first step for youths screened and triaged into low-intensity intervention.

## Methods

### Objectives

The primary aim of this randomized controlled study will be to evaluate the feasibility and acceptability of W-GenZD for adolescents with depression and/or anxiety in an outpatient children’s hospital system. Feasibility and acceptability will be assessed through self-report measures rating intervention feasibility, acceptability, and satisfaction, as well as study enrollment, app engagement, and group attendance. The secondary aim will be to compare the clinical impact of both the W-GenZD and skills group to reduce depression symptoms at 4 weeks end of treatment (EOT) relative to baseline (BL), with a corresponding hypothesis that W-GenZD will be noninferior to the skills group. As tertiary aims, therapeutic working alliance, mood, and anxiety levels in both groups will be assessed. Exploratory aims will include the observation of the utilization and performance of the safety procedures utilized within this study (please see below for a description of the safety net protocol and adverse event and serious adverse event assessment).

### Study Design

This study will randomize adolescents aged 13-17 years seeking treatment for depression and/or anxiety to the W-GenZD or CBT-group. The study will include 3 core assessments at screening/BL, EOT at week 4, and end of study (EOS) at week 8, plus 2 brief surveys to be completed 5 days and 2 weeks after randomization. The primary outcomes for feasibility and acceptability will be assessed at EOT; app engagement and group attendance metrics will be collected throughout the intervention period. Self-reported depression symptoms (secondary aim) will be collected via the 8-item Patient Health Questionnaire (PHQ-8) [29] at BL, week 2, EOT, and EOS. Therapeutic working alliance (tertiary aim) will be assessed at day 5 as well as at EOT. Mood and anxiety levels (tertiary aims) will be assessed via the Mood and Feelings Questionnaire (MFQ) [30] and Generalized Anxiety Disorder questionnaire (GAD-7) [31], respectively, at BL, EOT, and EOS. Exploratory outcomes, specifically the utilization of safety procedures will be collected throughout the study.

### Ethics Approval

This study was reviewed and approved by WIRB-Copernicus Group institutional review board on March 31, 2022 (#20221397). Subsequent modifications were implemented to refine eligibility criteria, enhance recruitment language, and provide further clarification within the informed consent forms.

### Study Population

#### Study Setting

The study site is a 206-bed, freestanding not-for-profit children’s hospital and the heart of an integrated pediatric health care system. Situated in the southeastern United States, the hospital serves a racially and economically diverse region that is home to approximately 500,000 youths younger than the age of 21 years. The outpatient practice’s stepped care model begins with a semistructured brief clinical needs assessment, which identifies unmet basic needs and the level of care needed. Youths who meet 1 or more of the following criteria indicate a higher level of needs and are fast-tracked to comprehensive assessment and individual treatment: have had suicidal ideation within the last 2 weeks or is a current risk to self/others, present symptoms of psychosis, meet criteria for an active eating disorder, have a history of mania, have a substance use disorder as the primary clinical problem, are at an imminent risk of expulsion, or are currently prescribed 4 or more psychotropic medications. Most patients with routine level of needs are offered low-intensity, telehealth skill-based group interventions while waiting for individual treatment. Patients who decline or are not appropriate for the specific groups are placed on a waitlist for individual treatment. The proposed study did not influence or change these prescreening criteria.

#### Recruitment

Youths with low-intensity needs who are not currently in therapy and who have not had recent medication changes are invited to learn more about the proposed study. Families receive information about the study through a flyer in a practice registration packet, the elevators, and waiting room. Caregivers of all children seen in the practice are invited to provide verbal or written consent for contact from the research team for more information about the study. Clinicians are encouraged to provide information about the study after the initial clinical needs assessment for youths who may be eligible. Study staff will reintroduce the study over the phone and assess the family’s (adolescent and parent/guardian) interest in participating.

In terms of waiting list management, participants in need of additional care are able to access individual treatment after the study concludes. If a waiting list opening becomes available during the study, participants are informed of this and can choose to accept it. If accepted, they are withdrawn from the study. If participants access treatment from another provider during the study, they are withdrawn from the study.

#### Inclusion and Exclusion Criteria

Youths eligible for participation in the proposed study must also meet all of the following study-specific inclusion criteria: (1) have a routine level of need based on a brief clinical needs assessment; (2) have a diagnosis of depression and/or anxiety; (3) be 13-17 years of age, inclusive; (4) be a US resident; (5) be able to read and write in English (adolescent and parent/guardian); (6) own or have reliable access to a smartphone and reliable Wi-Fi access or sufficient data to engage with assigned treatment condition for the duration of the study; (7) be on a stable dose of any psychiatric medications for at least 60 days; (8) not be currently engaged in psychotherapy; (9) be available and committed to engaging with the program and complete assessments for an 8-week duration; and (10) have a family member who can engage in the discussion of safety planning in the event of suicidal ideation.

**Figure 1:**
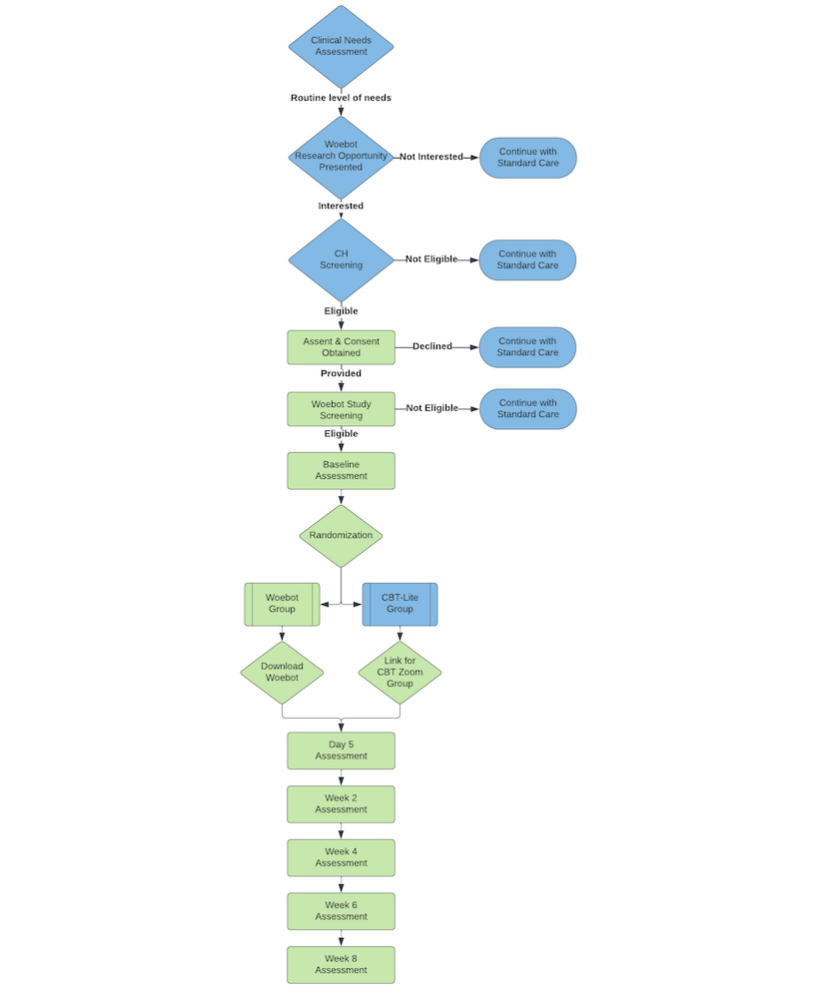
Participant flow through the study. CBT: cognitive behavioral therapy; CH: children’s hospital.

Youths who meet any of the following exclusion criteria will not be eligible for participation: (1) lifetime diagnosis of a psychotic disorder (including schizophrenia or schizoaffective disorder), bipolar disorder, autism spectrum disorder, or pervasive developmental disorder; (2) current diagnosis of developmental delay or intellectual disability; (3) reported suicidal ideation with a plan or intent or a suicide attempt within the past 12 months; (4) history of diagnosis of substance abuse within the past 12 months; (5) current use of benzodiazepines or certain sleep aids (zolpidem, eszopiclone, and zaleplon); (6) previous Woebot application use; and (7) enrollment of more than 1 member of the same household. Participants who meet all inclusion criteria and none of the exclusion criteria will complete BL assessments and be randomly assigned to a study treatment condition. Figure 1 shows the participant flow through the study.

#### Sample Size

The noninferiority analysis for comparing the 2 study conditions will be based on self-reported depressive symptom (PHQ-8) outcomes. In contrast, an exploratory analysis will be conducted on self-reported anxiety symptom (GAD-7) outcomes. In order to perform the noninferiority analysis, 260 participants (130 participants/arm) will be enrolled. Group sample sizes of 86 and 86 will achieve 81% power to detect noninferiority using a 1-sided, 2-sample *t* test. The margin of noninferiority is 2.0 [32]. The actual difference between the means is assumed to be –0.5. The significance level (α) of the test is .025. The data are drawn from samples with SDs of 5.3 and 4.3. Based on a sample size estimate of 172, the final sample size was set to 260 to account for 33% attrition (current recruitment status is provided in the *Results* section).

#### Randomization and Masking

Adolescents who meet inclusion criteria will be randomized 1:1 to the treatment groups. All clinical outcome measures are completed directly by participants accessing links to the measures in an electronic data capture platform. Neither participants nor staff are blinded to group assignment.

### Interventions

#### W-GenZD App

**Figure 2:**
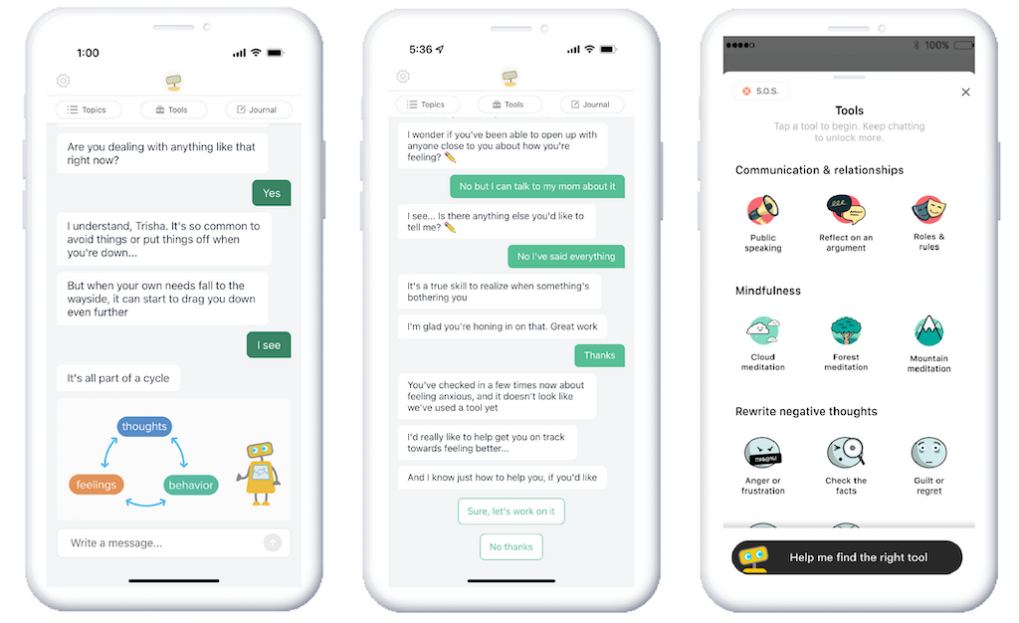
Screenshots of Woebot for Adolescents (W-GenZD), including examples of tools available to users.

W-GenZD is accessible as an iOS or Android app and is a relational agent–guided self-help program. It is only available to study participants using an access code for the investigational device. Using proprietary natural language processing and artificial intelligence, Woebot, the relational agent embedded in W-GenZD, guides users through a program that is personalized each day, in real time, to the user’s needs. Push notifications will prompt users to check in, and the cadence is based on the level of user activity (higher activity=higher cadence). The W-GenZD user experience is centered around mood and symptom tracking and goal-oriented conversations with Woebot. These conversations are tailored to the adolescent’s real-time report of the presenting situation, to help the adolescent develop emotion regulation skills in the context of their everyday life for the problem at hand, in their moment of need (see Figure 2). Users become familiar with Woebot as a friendly, helpful character that is explicitly not a human nor a therapist. Participants in the W-GenZD arm of the study will have access to W-GenZD for 4 weeks after randomization and are encouraged to use the app for a minimum of 5-10 minutes per day during the treatment period.

#### Safety Considerations

W-GenZD follows safety recommendations from the American Psychiatric Association [33] and American Medical Association [34]. W-GenZD’s safety features include providing the app’s privacy policy and terms of service, informing the user that they are talking to a robot and not a real person, stating that the app is not a crisis intervention for suicidal ideation, encouraging them to seek additional support if they are feeling unwell, and providing helplines for assistance. In addition, W-GenZD’s “safety net protocol” is based on a natural language processing algorithm that is designed to detect concerning language. The algorithm detects if a user inputs, via free-response text entry, concerning phrases or words that match an a priori identified and thorough list that may be used in crisis situations. When this occurs, the user is reminded that the app is not a crisis intervention service and helplines for additional support are offered.

For the purposes of this study, we have developed an escalation procedure that combines W-GenZD’s language detection capabilities with follow-up by trained clinicians. After the algorithm detects concerning language and if the adolescent confirms this, Woebot will let the adolescent know that someone on the hospital’s care team will be notified. W-GenZD will then send a Health Insurance Portability and Accountability Act (HIPAA)–compliant alert to designated clinical staff who will be on call to provide 24/7 follow-up of these messages. The licensed clinician on the study team will contact the adolescent, assess their safety and well-being, and refer to emergency services as necessary. Triage decisions resulting from the follow-up assessment will be logged separately, in accordance with the hospital’s standard procedures. Participants will continue in the study if they continue to meet the study inclusion criteria and do not require a higher level of care.

Study personnel may also learn of participant safety concerns in CBT-groups or by family or participant reports to the study team. In these instances, the follow-up procedure will be the same; the licensed clinician will contact the adolescent to assess their safety and well-being, with triage as needed and corresponding documentation.

#### Brief Telehealth-Delivered CBT-Group

The open CBT-groups are a rotating, 4-week intervention held once weekly for an hour. At least 2 groups are offered per week to allow the participants to attend the one that best fits their schedule. A licensed clinician facilitates each teletherapy group with up to 10 adolescents in each session. Group sessions begin with orientation and reviewing group rules, individual check-ins with each participant (rating stress level and hopefulness), followed by a guided mindfulness moment. Group attendees may choose to have their cameras on or off during group sessions but will be asked to have their cameras turned on for introductions and again at the very end of the session before they leave.

Groups are conducted on an open enrollment, rolling topic basis, allowing participants to join the next available session following randomization and complete all 4 sessions during their study treatment period. The 4 topics include: (1) building a coping toolbox, (2) accepting your feelings, (3) challenging negative thoughts, and (4) problem-solving.

The decision to use a 4-week CBT intervention was made to ensure that the duration of intervention of W-GenZD and CBT-group were equivalent. Both interventions are considered brief in duration.

### Measures

There will be 5 categories of measures that will be collected in this study: participant demographics and psychiatric history, feasibility and acceptability, clinical outcomes, safety data and engagement. Table 1 describes each category and its included measures, the source and content of data for each measure, and corresponding administration points. Participants complete assessments at BL, day 5, week 2, week 4 (EOT), and week 8 follow-up (EOS). After EOT, participants will no longer participate in the CBT-group nor have access to W-GenZD. Parents participate in telephonic check-ins at weeks 2, 4, 6, and 8 to identify changes in their child’s medications or logistical or safety concerns; if the latter is identified, the aforementioned safety follow-up protocol is initiated. Participants will receive reminders via email and SMS text messages for assessments with corresponding links to the surveys. Participants can be compensated up to US $125 in Amazon gift cards for the completion of all assessments.

**Table 1:** Assessment measures and administration schedule.

Assessment type and measure		Description	Administration
**Participant demographics and psychiatric history**			
	Survey questions	Demographic: gender and sexual identity, racial and ethnic background, highest level of education completed, etc Psychiatric history: psychiatric diagnoses and any prior or concomitant medications and prior therapy	BL^a^
**Feasibility and acceptance**			
	Client Satisfaction Questionnaire [35]	8-item measure used to assess client’s satisfaction with treatment on a 4-point scale (1=“very dissatisfied” to 4=“very satisfied”) [35]	EOT^b^
	Satisfaction Questionnaire	3 open-ended questions about what they found most helpful about their treatment, what would make it better, and if there is any other feedback they would like to share	EOT
	URPI^c^-Feasibility and URPI-Acceptability (from the URPI-Revised scale [36])	6-item subscales inquire about factors that impact treatment usage (ie, intervention quality)	BL and EOT
**Clinical outcomes**			
	PHQ^d^ [29]	Abbreviated version of the PHQ-9, the PHQ-8, will be used to assess mood and anxiety symptoms	BL, week 2, EOT, and EOS^e^
	GAD^f^ [31]	Self-report tool to assess the frequency and severity of anxious thoughts and behaviors over the past 2 weeks; it is a widely utilized, reliable, and valid measure of anxiety	BL, EOT, and EOS
	MFQ^g^ [30]	Short version is a child self-report assessment consisting of a series of 13 descriptive phrases related to the participant’s recent feelings and behaviors	BL, EOT, and EOS
	WAI-SR^h^ [37]	12-item Working Alliance Inventory–Short Revised version with minor changes to language, replacing “therapist” with the name of the assigned treatment group measure of therapeutic bond; consists of a composite score and 3 subscales: Bond, Goal, and Task	Day 5 and EOT
**Safety data**			
	Incident reports	Utilization data, including frequency, type, and outcomes to report on incidents of “safety net protocol” triggers and the occurrence and relatedness of adverse events or serious adverse events	Throughout study
**Engagement**			
	W-GenZD^i^ use and CBT-group^j^ attendance	W-GenZD: days in app and content engagement CBT-group attendance	Throughout treatment until EOT

^a^BL: baseline.

^b^EOT: end of treatment.

^c^URPI: Usage Rating Profile–Intervention.

^d^PHQ: Patient Health Questionnaire.

^e^EOS: end of study.

^f^GAD: Generalized Anxiety Disorder.

^g^MFQ: Mood and Feelings Questionnaire.

^h^WAI-SR: Working Alliance Inventory.

^i^W-GenZD: Woebot for Adolescents.

^j^CBT-group: cognitive behavioral therapy–based skills group.

### Data Collection

All study data, from participant self-report and on-site study personnel, are gathered through a HIPAA-compliant survey and electronic data capture platform. All data obtained via the mobile app will be encrypted and stored on Amazon Web Services and/or Google Cloud Platform; Woebot Health data-gathering and storage procedures are compliant with both HIPAA and the European Union’s General Data Protection Regulation. Only members of the research team will have access to identifiable study data. Deidentified data will be accessible to the product development team (designers, user researchers, and product managers) as well as the research team.

### Statistical Analysis

Data will be reported per treatment arm and overall, for all time points in the study. For continuous variables, means and SDs will be reported if the data are normally distributed; for nonnormal data, medians and IQRs will be reported. For categorical variables, frequencies and percentages will be reported.

To assess the primary aim (feasibility and acceptability of W-GenZD), the descriptive statistics to be reported include the Usage Rating Profile–Intervention (URPI), Client Satisfaction Questionnaire, Satisfaction Questionnaire, and URPI-Feasibility and URPI-Acceptability scales, as well as app engagement and group attendance metrics. For the secondary aim (comparison of the clinical impact of W-GenZD and the skills group to reduce depression symptoms), a test of noninferiority (1-sided) will be used to determine if EOT PHQ-8 change scores for W-GenZD are noninferior to the CBT-group. α will be set to .025. CIs will be reported for the EOT clinical outcomes. The main analyses will be conducted within the intent-to-treat group and with a sensitivity analysis in the per-protocol group. If the data are nonnormally distributed, a suitable nonparametric model will be used. For the tertiary aim (assessment of therapeutic working alliance, mood, and anxiety levels in both groups), descriptive statistics will be reported for the Working Alliance Inventory–Short Revised version, GAD-7, and MFQ at all time points.

Additional sensitivity analysis will be performed. Although randomization should ensure the equality of the 2 groups at BL, tests of potential between-group differences will be conducted. If differences are found, a regression-based approach will be used for the noninferiority test-based CIs. Any BL covariate where a significant difference between the 2 treatment groups was achieved would be input into the model. If the model is a poor fit, a suitable nonparametric alternative will be selected. All statistical analyses will be performed using R statistical software (R Foundation for Statistical Computing).

## Results

Recruitment began in May 2022. At the time of submitting this paper for review (December 8, 2022), we have randomized 133 participants. The study’s a priori planned end of recruitment date was November 30, 2022, which has been extended to December 16, 2022, to allow for those presently in the queue in the children’s hospital screening process to be assented/consented and screened for study eligibility. Final enrollment numbers will be reported in the outcome’s manuscript.

## Discussion

### Significance of Study

This paper describes the rationale, design, and corresponding methodology for testing the feasibility and acceptability of W-GenZD, which includes Woebot, a relational agent that offers a CBT-guided self-help program delivered through a smartphone app. Embedding the study within a MH treatment environment, a children’s hospital’s outpatient MH clinic, allows for learnings specific to real-world clinical practice settings. In this study, adolescents diagnosed with depression and/or anxiety and who are treatment seeking will be randomized to 1 of 2 technology-leveraged treatments, W-GenZD or CBT-group, both of which are potential “first step” interventions within a stepped care treatment paradigm. Participants in the study must meet both existing criteria for participation in a low-intensity treatment as well as the study-specific inclusion and exclusion criteria. Thus, enrolled youths randomized in the study are representative of treatment-seeking youths with depression and anxiety within this hospital’s community. This study will also evaluate if the outcomes yielded by the W-GenZD and brief telehealth-delivered CBT-group are analogous. We anticipate that W-GenZD will be noninferior to the CBT-group in reducing adolescent depression (PHQ-8) at 4 weeks. We will also explore self-reported mood (MFQ) and anxiety symptom levels (GAD-7) in both treatment groups as a tertiary aim.

Working alliance has been long identified and studied as an important construct in the psychotherapeutic literature, including across treatment modalities and theoretical foundations. Not only has it been associated with positive treatment outcomes, but it also holds face validity as a meaningful manifestation of a productive and aligned therapeutic relationship. Previous data demonstrated Woebot’s ability to establish and sustain working alliance at levels analogous to human-delivered CBT interventions [20]. The inclusion of working alliance in this study allows for further exploration of this construct as it applies to a sample of treatment-seeking adolescents diagnosed with depression and/or anxiety engaged in a technology-supported MH intervention. By evaluating therapeutic alliance levels in both study conditions, we can gain a greater understanding of therapeutic bonding in both a relational agent and a telehealth-delivered brief CBT-group, with the latter being a more relatively familiar group treatment modality. In future work, the potential relationship of therapeutic alliance to improvement in symptoms among adolescents can be explored.

This protocol integrates a real-world safety assessment into the use of W-GenZD as well as the CBT-group to ensure a safety net is available to adolescents who may express ideation of self-harm. This allows the study of responses to safety concerns with a vulnerable research population receiving either a relational agent (W-GenZD) intervention or a telehealth-delivered group CBT intervention. The application and refinement of this protocol may provide an opportunity for the expansion of real-world digital MH intervention to other adolescent clinical populations.

This study goes beyond self-report measures alone and will invite perspectives from both caregivers and adolescents. This multireporter approach is critical in providing multiple perspectives about the adolescent’s functioning. It is consistent with real-world clinical practice, in which the youth’s voice is a primary source of clinical information and family perspectives are integrated into the formulation [38].

### Limitations

Study participants include treatment-seeking youths who present alongside their parent/guardian’s request for MH care and therefore may not be representative of non–treatment-seeking symptomatic youths in the community. For safety reasons, our study excluded youths with high-acuity symptoms such as active suicidal ideation and bipolar disorder. As a result, findings may not be generalizable to youths with more acute symptoms and treatment needs. In addition, in a study of depression and anxiety, group therapy as a treatment arm may limit the representativeness of the sample due to the potential discomfort of some young people with a group modality. Finally, to ensure equal durations of intervention, the group therapy intervention was designed as a brief course of treatment, which may be less representative of typical longer courses of group CBT treatment.

### Conclusion

We have described a randomized controlled trial that seeks to establish the feasibility and acceptability of a relational agent digitally delivering CBT within a pediatric outpatient MH hospital setting. Demonstrating W-GenZD as noninferior to a CBT-group may have implications for the expansion of options for adolescents seeking MH treatment for depression and/or anxiety. Providing technology-leveraged effective treatment options for those at lower-acuity levels may help reserve limited resources for those with more severe MH needs.

## Data Availability

The data sets underlying the results of this study are confidential and include proprietary commercial information. Requests can be submitted to the corresponding author and will be subject to review and approval by the data owner.

## Abbreviations

BL: baseline
EOS: end of study
EOT: end of treatment
GAD-7: Generalized Anxiety Disorder
HIPAA: Health Insurance Portability and Accountability Act
MFQ: Mood and Feelings Questionnaire
MH: mental health
PHQ-8: Patient Health Questionnaire
URPI: Usage Rating Profile-Intervention
W-GenZD: Woebot for Adolescents
